# Mandatory food fortification in the eastern Mediterranean region results in reduced prevalence of neural tube defects

**DOI:** 10.3389/fpubh.2025.1664607

**Published:** 2026-01-12

**Authors:** Sylvia Roozen, Quentin Johnson, Eman Ibrahim, Salima Al Maamari, Amina Barkat, Saleh Al Shammakhi, Fatima Zohra Laamiri, Aguenaou Hassan, Hasnae Gamih, Abdelhakim Yahyane, Ayoub Al Jawaldeh

**Affiliations:** 1 International Federation for Spina Bifida and Hydrocephalus, Brussel, Belgium; 2 Governor Kremers Centre-Maastricht University Medical Centre, Maastricht, Netherlands; 3 Regional Office for the Eastern Mediterranean (EMRO), World Health Organization (WHO), Cairo, Egypt; 4 Nutrition Department, Ministry of Health, Muscat, Oman; 5 Faculty of Medicine and Pharmacy of Rabat, Mohamed V University Rabat, Rabat, Morocco; 6 University Hassan I_Settat, Settat, Morocco; 7 Université Ibn Tofail, Faculty of Sciences, Kenitra, Morocco; 8 Ministry of Health, Directorate of Public Health, Rabat, Morocco

**Keywords:** neural tube defects, spina bifida, folic acid, preconceptional supplementation, pregnant women, women of reproductive age, nutrition-specific interventions, food fortification

## Abstract

**Introduction:**

Neural Tube Defects (NTDs) are preventable congenital malformations affecting the brain and spinal cord that occur very early in pregnancy, often before gestation is recognized. Globally, at least 20 per 10,000 live births are estimated to be affected by NTDs, although the true prevalence is likely higher due to unreported miscarriages, elective terminations, and gaps in surveillance systems. Mandatory fortification of staple foods with folic acid (Vitamin B9) is widely recognized as the most effective and cost-efficient strategy to reduce the prevalence of NTDs, whereas voluntary dietary supplementation programs have demonstrated limited uptake and population-level impact.

**Methods:**

This study employs a situational analysis of mandatory food fortification policies and programs in the Eastern Mediterranean Region. Available epidemiological data on NTD prevalence were reviewed alongside evidence on folic acid fortification effectiveness and program implementation. The analysis also considered the role of surveillance systems, program coverage, and multisectoral engagement across public, private, and civic sectors to assess feasibility, sustainability, and health impact.

**Results:**

Estimates of the impact of mandatory food fortification programs in the Eastern Mediterranean Region are constrained by heterogeneous data sources, weak or incomplete surveillance systems, and variable program coverage between and within countries. While countries with established fortification policies show potential reductions in NTD prevalence, inconsistent implementation and monitoring limit the ability to accurately measure outcomes. Insufficient coordination among stakeholders further affects program effectiveness and long-term sustainability.

**Discussion:**

Despite strong evidence supporting mandatory folic acid fortification as an effective NTD prevention strategy, significant implementation and monitoring challenges persist in the Eastern Mediterranean Region. Strengthening surveillance systems, improving data quality, and enhancing multisectoral collaboration are critical to translating policy commitments into measurable health outcomes. By integrating epidemiological evidence with implementation insights, this analysis identifies priority areas for improving regional fortification efforts and contributes to broader global strategies aimed at reducing the burden of NTDs.

## Introduction

Neural Tube Defects (NTDs) are the second most common type of structural birth defects affecting communities worldwide. NTDs are an umbrella term that includes anencephaly, encephalocele, spina bifida, and their combinations, along with possible secondary impairments such as hydrocephalus and neurogenic bowel and bladder dysfunction (1). Globally, at least 20 per 10,000 live births are affected by NTDs. It is likely to be an underestimate, due to limitations of surveillance systems, underreporting, unaccounted stillbirths, and early pregnancy terminations (2, 3). The NTD prevalence estimates are reported to be higher in low- and middle-income countries, where factors such as inadequate nutrition, limited access to healthcare, and suboptimal prenatal care contribute to increased risks, resulting in preventable morbidity and mortality (4).

Evidence from prevention research has demonstrated the critical role of the intake of folic acid (Vitamin B9) during the preconception period. In particular, countries that have implemented mandatory fortification of staple foods with folic acid, often combined with iron, have reported reductions in the prevalence of spina bifida and other NTDs ranging from 30% to 70%, as well as decreases in iron deficiency anemia (4–6).

Global advocacy and technical guidance from WHO and other UN agencies have been instrumental in the promotion of food fortification policies. For instance, the WHO recommendations on fortifying wheat and maize flour with iron and folic acid provide guidance and technical support for countries to implement prevention programs (7). Also, regional evaluations, such as the WHO report on wheat flour fortification in the Eastern Mediterranean Region, highlight both progress as well as challenges in achieving optimal coverage and compliance (8). In addition, evidence from implementation science demonstrates that the successful implementation of fortification programs require equal and coordinated partnerships among public, private and civic sectors to ensure feasibility, sustainability, and measurable health outcomes (9, 10).

Decades of advocacy for mandatory food fortification led to the adoption of the WHA76.19 Resolution, titled “Accelerating Efforts for Preventing Micronutrient Deficiencies and Their Consequences, Including Spina Bifida and Other Neural Tube Defects, through Safe and Effective Food Fortification” (11). This resolution signifies a significant global commitment for the prevention of micronutrient deficiencies and their associated health consequences.

Despite global evidence supporting the effectiveness of folic acid fortification, systematic assessments of mandatory food fortification programs in the EMR remain scarce. Regional data are limited, heterogeneous, and often constrained by weak surveillance systems, making it challenging to evaluate program coverage, compliance, and impact on NTD prevalence (8). This study aims to conduct a descriptive situational analysis of mandatory food fortification in the EMR, quantifying its impact on NTD prevalence and identifying actionable strategies to enhance program effectiveness.

## Methods

A literature search was conducted in PubMeD, Global Health Observatory (GHO), the Global Database on the Implementation of Nutrition Action (GINA), WHO EMRO (Regional Office for the Eastern Mediterranean) website, and governmental websites. Additional data was also explored using the Global Fortification Data Exchange (GFDx) tool. The query consisted of keywords and their synonyms related to Neural Tube Defects, prevalence, EMR, folic acid, and food fortification. An additional illustration of the impact of mandatory food fortification will be provided by two country reports Oman and Morocco.

For the NTD prevalence estimates in Oman, statistics were obtained from morbidity data from all outpatient clinics and hospitals through Al Shifa Health System, as reported in the Ministry of Health Annual Health Statistics (12). Data were included if they reported primary morbidity cases of NTDs between 2014 and 2023 and were identified using ICD-10 or ICD-11 codes: Q00–Q00.2 (Anencephaly), Q01–Q01.9 (Encephalocele), and Q05.0–Q05.9 (Spina Bifida), including all subcategories. Records were excluded if they lacked ICD coding, did not report primary morbidity data, fell outside the defined period or region, or represented duplicates due to patient referrals between facilities. This dataset was linked to available historical data from 1991. Hospital data before 1997 were recorded using the basic tabulation list of ICD-9, with combined codes for spina bifida and hydrocephalus (741) and (742.3) while data after 1997 were recorded using ICD-10 codes (13). For the presentation of the prevalence estimates of NTDs in Morocco, data from a multicenter study (from January 2012 to December 2022) at 20 public hospitals in Morocco covering the following regions was included: Oued Eddahab-Lagouira, Laayoune-Boujdour-Sakia Lhamra, Guelmim-Smara, Souss-MassaDarâa, El Gharb-Chrarda-Bni Hssen, Chaouia-Ouardigha, Marrakech-Tensift-El Haouz, Oriental, Grand Casablanca, Rabat-Salé-Zemmour-Zaër, Doukkala-Abda, Tadla-Azilal, Meknes-Tafilalet, Fes-Boulemane, Taza-Al Hoceima-Taounate, and finally Tangier-Tetouan. Data included all women whose fetus or newborn showed an isolated or combined neural tube defect, whether diagnosed through a prenatal ultrasound or the systematic clinical exam at birth, regardless of the pregnancy term or outcome.

In the presented data included were all live births, born in one of the medical structures concerned during the study period and having at least one abnormality of closure of the clinically visible neural tube isolated or associated with a congenital malformation as well as the oro-facial clefts. NTDs were divided into two groups: (1) the spina bifida group including open spinal dysraphism (meningocele, myelomeningocele) and spina bifida occulta; (2) the anencephaly group defined by the absence of a cranial vault with total or partial absence of the brain. The oro-facial clefts were divided into three groups: (1) cleft palates, (2) cleft lip, and (3) cleft lip and palate labiopalatal.

Ethical approval was not required for this study, as it involved a retrospective analysis of de-identified, routinely collected hospital data, in line with WHO guidance on secondary use of anonymized health information.

## Current status of fortification of staple foods in the EMR

### Food fortification and micronutrient supplementation in the EMR

Fortification is an important public health intervention to improve micronutrient status. Fortification of industrially processed wheat flour when appropriately implemented is a simple, inexpensive and effective strategy for supplying vitamins and minerals. The WHO Regional Office for the Eastern Mediterranean together with international partners launched an initiative in 1999 which led to that nearly all the countries of the Region by 2022 are fortifying wheat flour with at least iron and folic acid. Currently, wheat flour fortification is widely used in countries of the Eastern Mediterranean Region through voluntary and mandatory regulations (15 countries). There are seven countries in the Region who are not implementing wheat flour fortification (Egypt, Lebanon, Pakistan, Syria, Libya, Somalia, and Tunisia). Qatar and UAE fortify more than half of their industrially milled wheat flour even though it is not mandatory. In 1996, Oman was the first country to fortify flour with folic acid on national scale to prevent fetal birth defects (14).

Flour fortification is applied through voluntary or mandatory regulations in 15 countries and territories of the Eastern Mediterranean Region. Flour fortification is mandatory in 10 countries and territories of the Region. Despite the Challenges, Egypt's Ministry of Supply and Internal Trade (MOSIT) continues to partner with the Food Fortification Initiative (FFI) to restart the country's wheat flour fortification program, which ended in 2014 (14, 15). Iron and folic acid are the most common nutrients used to fortify wheat flour in the Region. Iron is added to wheat flour in 14 countries: Afghanistan, Bahrain, Djibouti, Iraq, Jordan, Kuwait, Morocco, Palestine, Oman, Qatar, Saudi Arabia, Sudan, United Arab Emirates, and Yemen. Folic acid is sadded to wheat flour in 13 countries: Afghanistan, Bahrain, Iraq, Jordan, Kuwait, Morocco, Palestine, Oman, Qatar, Saudi Arabia, Sudan, United Arab Emirates and Yemen. Fortification with B12 vitamins occurs in several countries, including Afghanistan, Djibouti, Jordan, Oman, Palestine and Sudan. Vitamin A fortification happens in three countries: Jordan, Palestine and Sudan. A total of five countries fortified with zinc: Afghanistan, Djibouti, Jordan, Palestine, and Sudan. Vitamin D fortification takes place only in Jordan, Kuwait, Oman, Palestine, Qatar, Saudi Arabia, and United Arab Emirates. Wheat flour fortification has contributed to progress in tackling micronutrient deficiencies in the Region, but further progress is needed. Many countries need to review their policies and practices to ensure that best practice guidance on wheat flour fortification is being implemented. Only Oman currently has a standard for oil fortification (a voluntary standard for fortification with vitamins A and D), see also Tables 1, 2.

**Table 1:** Overview of the milling industry and fortification in EMR.

**Country**	**Number of industrial mills (>20 metric tons/day)**	**% Flour/rice produced in industrial mills**			**% Industrially milled flour/rice that is fortified**
	**Wheat**	**Wheat**	**Maize**	**Rice**	**Wheat**
Afghanistan	27	81	**–**	79	71.2
Bahrain	1	100	100	100	90
Djibouti	1	100	100	100	**–**
Egypt^*^	162	90	0	100	0
Iran	100	100	0	78	100
Iraq	300	100	3	97	0
Jordan	22	100	100	100	93
Kuwait	1	100	**–**	100	100
Lebanon	8	100	**–**	100	0
Libya	1	100	**–**	100	0
Morocco	137	100	97	89	27
Oman	2	100	1	100	89
Pakistan	1080	32	0	90	5
Palestine	**–**	100	100	100	100
Qatar	1	100	**–**	100	90
Saudi Arabia	9	100	100	100	100
Somalia	1	100	100	100	**–**
Sudan	25	80	76	84	40
Syria	57	82	**–**	100	5
Tunisia	28	100	100	100	0
UAE	4	100	90	100	90
Yemen	6	100	3	94	100

This table presents data from the FFI database. The industrial wheat and maize flour mill include the capacity to mill more than 20 metric tons/day. For the industrial rice mill, this includes the capacity to mill at least 5 metric tons/hour of paddy rice. Not shown in the table are the number of industrial rice mills (>20 metric tons/day) for Afghanistan (2) and Pakistan (200). Also not shown is the number of % industrially milled maize that is fortified in Sudan (25). Estimates may differ from Global Fortification Data Exchange estimates as FFI assumes imported flour/rice is industrially milled. FFI includes imported flour/rice into the total percentage of flour/rice produced in industrial mills and percentage of industrially milled flour/rice that is fortified.

^*^Despite the Challenges, Egypt's Ministry of Supply and Internal Trade (MOSIT) continues to partner with the Food Fortification Initiative (FFI) to restart the country's wheat flour fortification program, which ended in 2014.

**Table 2:** Status of flour fortification in the EMR.

**Country**	**Fortification status**	**Iron ppm**	**Zinc ppm**	**Folic acid ppm**	**B12 ppm**	**Other vitamins ppm**	**Estimated coverage (Flour produced in industrial mills)**	**NTD rates Per 10,000 Livebirths**
Afghanistan	Mandatory	15 NaFeEDTA	50 ZnO	1	0.008	–	81%	32
Bahrain	Mandatory	60 Reduced Iron	–	1.5	–	–	100%	17
Djibouti	Mandatory	60 Fe Electrolytic Iron	40 ZnO	–	1.3	–	100%	22
Egypt^*^	None		–		–	–	90%	17
Iran	Mandatory	–	–	–	–	–	–	–
Iraq	Voluntary	45 Ferous Sulfate (FeSO4)	–	2.1	–	–	100%	18
Jordan^**^	Mandatory	33.97 Ferous Sulfate (FeSO4)	20.08	1.5	0.008	B1,2,3 Calcium, 14.15 B6, 3.6 Niacin, 35 Riboflavin, 3.6 Thiamin, 2.9 Vit A, 1.5 Vit D, 0.0145	100%	33
Kuwait	Voluntary	30 Fe	–	1.75	–	B1,2,3 Calcium, 2115 Niacin, 52.91 Riboflavin, 3.96 Thiamin, 6.38 Vit D, 0.0137	100%	17
Lebanon	None	–	–	–	–	–	100%	–
Libya	None	–	–	–	–	–	100%	–
Morocco	Mandatory	10.4 NaFeEDTA	–	1.0	–	–	100%	22
Oman	Mandatory	60 Fe Electrolytic iron, Ferrous sulfate (FeSO4) 120^**^	–	1.5	–	B12^***^ Vit D^***^	100%	12
Pakistan	None	–	–	–	–	–	–	32
Palestine	Mandatory	34.4 Ferrous sulfate (FeSO4)	20.6	1.5	0.004	B1, 2, 3 B6, 3.6 Niacin, 35 Riboflavin, 3.6 Thiamin, 2.9 Vit A, 1.5 Vit D 0.023	100%	55
Qatar	Voluntary	30 Ferrous sulfate (FeSO4)	–	1.75	–	B1,2,3, D Calcium, 2115 Niacin, 52.91 Riboflavin, 3.96 Thiamin, 6.38 Vit D, 0.0137	100%	17
Saudi Arabia	Voluntary	30 Fe	–	1.75	–	B1,2,3, D Calcium, 2115 Niacin, 52.91 Riboflavin, 3.96 Thiamin, 6.38 Vit D, 0.0137	100%	17
Somalia	None	–	–	–	–	–	100%	22
Sudan^**^	Voluntary	30 Ferrous sulfate (FeSO4) 20 NaFeEDTA 60 Fe Electrolytic iron 30 Ferrous fumarate	–	1.3	0.01	1.5 Vitamin A^**^	80%	22
Syria	None	–	–	–	–	–	82%	18
Tunisia	None	–	–	–	–	–	100%	–
UAE	Voluntary	30 Fe	–	1.75	–	B1,2,3, D Calcium, 2115 Niacin, 52.91 Riboflavin, 3.96 Thiamin, 6.38 Vit D, 0.0137	100%	17
Yemen	Mandatory	60 Reduced Iron	–	1.5	–		100%	17

This table presents nutrition data from the FFI database, Global Fortification Data Exchange. All reported nutrients are expressed in parts per million (ppm). For Calcium, Iron, Type of Iron, Folic acid, Zinc, B6, B12, Niacin B3, Rioboflavin B2, and Thiamin B1. Also, Vitamin A and Vitamin D. The zinc deficiency estimates for different countries in the region could be found in WHO-Micronutrient database (34).

This table presents prevalence estimates extracted from Blencowe et al. (2). This figure may not include pregnancy loss or terminations of pregnancies due to prenatal diagnosis of a neural tube defect. With all folic acid interventions in place, the birth prevalence of neural tube defects would be about 6 per 10,000.

Estimates may differ from Global Fortification Data Exchange estimates as FFI assumes imported flour/rice is industrially milled. FFI includes imported flour/rice into the total percentage of flour/rice produced in industrial mills and percentage of industrially milled flour/rice that is fortified.

^*^Despite the Challenges, Egypt's Ministry of Supply and Internal Trade (MOSIT) continues to partner with the Food Fortification Initiative (FFI) to restart the country's wheat flour fortification program, which ended in 2014. ^**^Data obtained only from Global Fortification Data Exchange. ^***^Data obtained from https://qanoon.om/p/2022/mafw20220185/.

As Table 1 illustrates (data source: FFI database; see table note for definitions), Iraq has one of the largest milling capacities in the EMR (300 industrial mills, 100% wheat industrially milled). However, none of the flour is fortified, highlighting a major gap between infrastructure and implementation. Only 27% of flour is fortified in Morocco, despite full industrial milling (100%), which reflects missed opportunities for public health impact. On the other hand, Oman's concentrated sector, which consists of only 2 industrial mills covering all wheat, facilitates enforcement and has achieved 89% coverage. Pakistan illustrates a different structural challenge: although it has the largest number of mills (more than 1,000, nearly 1,080), only 32% of wheat is industrially milled and just 5% fortified, notably limiting program reach. These differences highlight the critical role of the milling industry in determining the success of the fortification programs and indicate the need to consider it alongside legislation when evaluating outcomes. Similarly, the case of Kuwait shows how a single industrial mill facilitates enforcement and ensures full coverage, in contrast with a greater and more fragmented milling sectors for instance Pakistan's. In the UAE and Qatar, a significant proportion of flour is produced in industrial mills (90% and 100%, respectively) affords favorable conditions for fortification programs, which explains their high coverage rates.

Table 2 illustrates that per capita consumption of wheat flour presents significant variation among EMR countries, reflecting diverse dietary patterns and levels of reliance on wheat-based staples. Tunisia, Syria, and Morocco demonstrate the greatest per capita wheat flour consumption ranging from 128–148 kilograms per person annually (kg/pp/annum), with daily wheat availability exceeding 350 grams per capita. This elevated consumption underscores the pivotal importance and role of wheat in the nutritious diet and the possible population-wide impact of fortification programs in these situations.

Inversely, countries such as Qatar and Oman have much lower wheat flour consumption levels approximately 10–50 kilograms per person annually (kg/pp/annum). However, their food supply chains are highly centralized and dominated by imported, industrially milled flour. This structure supports the implementation of quality control and contributes to the achievement of high fortification coverage despite lower overall intake.

Intermediate consumption countries, which include Kuwait, Jordan, KSA, Pakistan and Sudan have moderate daily wheat availability (ranging approximately between 150 and 220 grams per capita per day (g/c/d), as presented in Table 2), suggesting opportunities for effective and efficient fortification implementation provided that enforcement and supply consistency are sustained.

Overall, these variations emphasize the significance of both dietary dependences on wheat and structural the characteristics of the milling and import systems jointly determine the effectiveness and potential reach of flour fortification programs in the EMR countries.

WHO-EMRO has been collaborating with the Ministry of Health to reintroduce flour fortification in the region, but progress has been slow, particularly in Lebanon and Tunisia, where there has been little interest in implementing flour fortification. This lack of engagement has hindered the advancement of the food fortification program.

Table 2 provides an overview of the status of folic acid fortification and the most recent data on NTD rates in some countries. Not all countries in the region have specific data on NTDs. In Saudi Arabia, Oman, and Iran, the decrease in neural tube defects per 1,000 births following the introduction of folic acid fortification in flour was 60%, 70%, and 31%, respectively, according to the FFI. The prevalence of NTDs before fortification in these countries was as follows: Saudi Arabia (1.9), Oman (0.96), and Iran (3.16). After fortification, the prevalence was reduced to: Saudi Arabia (0.76), Oman (3.17), and Iran (2.19) (16).

Countries such as Lebanon, Libya, Syria, Somalia, Egypt, Pakistan, and Tunisia currently lack national standards for flour fortification. However, these nations could greatly benefit from implementing flour fortification programs.

## Food fortification programs in practice in the region

Wheat is the most widely consumed cereal in the region, forming a major part of the diet for nearly the entire population. A significant challenge for countries in the region is their reliance on imported wheat from key exporting countries, which include Australia, Canada, the European Union, Russia, Ukraine, and the USA. Table 3 offers an overview of the daily availability of wheat in each country within the EMR.

**Table 3:** Overview of daily food availability of wheat as of 2025 in the EMR.

**Country**	**Daily food availability of wheat (g/c/d)**
Afghanistan	364
Bahrain	131
Djibouti	215
Egypt	305
Jordan	224
Kuwait	191
Lebanon	235
Libya	235
Morocco	388
Oman^*^	33
Pakistan	254
Qatar	137
Saudi Arabia	215
Sudan	131
Syria	311
Tunisia	415
UAE	209
Yemen	251

Information based on FFI Country data webpages https://www.ffinetwork.org/country-profiles. The amount of flour or rice available in food supply is expressed in grams/person/day. Flour data is based on typical extraction rate of 75%. ^*^Data obtained from FAO 2021.

Several key factors impact fortification programs in the region: (i) The importation of premix feeders from overseas suppliers based in Europe, China, India, and the USA; (ii) The importation of premix powders for flour fortification, with some suppliers offering both fortification premix and flour improvers; (iii) Many countries in the region have reduced or eliminated import duties on equipment and premixes, lowering financial barriers for the importation of these materials. In some cases, the Ministry of Health imports the premix duty-free from overseas suppliers.

The milling industry in the Middle East plays a crucial role in supporting national initiatives aimed at addressing micronutrient deficiencies. Wheat is regarded as the primary staple food and is the most suitable vehicle for fortification in the region.

Since wheat production is limited in the Eastern Mediterranean Region (EMR), most wheat grain is imported from countries such as Australia, Canada, the European Union, Kazakhstan, Russia, Ukraine, and the USA, and then milled within the importing country.

While there is limited available data on flour exports, it is important to note that some of the wheat milled into flour in Saudi Arabia and the UAE is exported to other countries outside their borders. This highlights the potential for fortified flour to impact broader regional nutrition outcomes, depending on the standards and policies applied in both importing and exporting nations.

### Premix and premix suppliers

Premix for cereal flour fortification typically contains two to six micronutrients. The WHO recommends that countries seeking to add micronutrients to staple foods, such as wheat flour, maize flour, salt, and vegetable oil, should follow the applicable WHO guidelines for each food type, considering the typical daily consumption of these staples.

In many countries that fortify staple foods, premix is imported from suppliers in China, Europe, India, and the USA. To facilitate food fortification efforts, the WHO advises that premix and fortification equipment should be exempt from or subject to minimal duties and import taxes (16–18).

Trade constraints on imported wheat, such as the ongoing war in Ukraine, have led to high global wheat prices due to disruptions in supply and increased demand from wheat-producing nations worldwide.

To provide a clearer picture of the current status of flour fortification, this paper will now examine case studies from Oman and Morocco.

## Examples of evolution and current status of flour fortification

### Sultanate of Oman

Sultanate of Oman is located in West Asia on the south eastern coast of the Arabian Peninsula with a population of 5,163,173 (19). The country is classified as high-income country with a GDP per capita reaching 25,056.8 US $. The primary health care witnessed remarkable improvements over the last 40 years and started fighting malnutrition of all forms including micronutrients deficiency very early. A national study in 1986 reported that 54% of pregnant women in Oman had hemoglobin (Hb) values less than 11 g/dl. As a result of the study, a national supplementation programme was started in 1990 to provide all pregnant women with a daily dose of 200 mg iron and 5 mg folic acid. However, 48.5% of Omani pregnant women were still anemic in 1993 (13, 20).

While the supplementation programme continues, in 1996 the Ministry of Commerce, based on the request of the Ministry of Health, mandated the fortification of white flour with 30 ppm iron as elemental (electrolytic) iron and 1.5 mg/kg folic acid and the Oman Flour Mills complied with the legislation. Population coverage was expected to be high as almost 80% of the flour was supplied by one mill. Oman Flour Mills (OFM) was the only mill in the country and supplied more than 80% of the flour and bread demand with 800MT/day capacity. Main types of flour produced were the atta flour with high extraction rate of 87% and accounted for 60% of the production in 1996; and low extraction rate flour that accounted to 40% of the production (21). Fiber content of high extraction flour inhibits iron absorption therefore it was decided to fortify the white flour only (20). Oman was the first country in the middle east to make fortification of flour with folic acid a national compulsory legislation. Household income and expenditure data from Oman were used to estimate the average food consumption for a household for a year (May 1999 to May 2000). The estimates showed that without food fortification the average folic acid intake of the Omani population was only 123 μg, 33% of the per capita requirements, based on average energy requirement levels of 2,100 kcal and folate density goals of 150–200 μg per 1,000 kcal. With fortification, the estimated average per capita folate level in the diet would increase to 489 μg (122% of the target) for an energy consumption of 2,100 kcal. By 2004 the household coverage of fortified flour and products in Oman was 81% (13). This indicates the high compliance of the mills to the regulation of mandatory fortification. Also, Oman is more likely to achieve a wider distribution of fortified flour from the large mills and therefore the impact is higher compared to for instance Morocco.

### Kingdom of Morocco

Morocco chose in 2001 the enrichment of soft wheat flour to combat disorders due to micronutrient deficiencies. The choice of flour as a vehicle for enrichment is based on its large and high consumption by the Moroccan population (about 364 g/person/day in 2000), as well as on the technical feasibility of enrichment. The enrichment process began in 2005 with the addition of 45 mg of iron, in the form of electrolytic elemental iron, per kilogram (kg) of flour. The iron was incorporated into a prepared mixture comprising vitamins B1 (4.5 mg/kg), B2 (2.79 mg/kg), PP (36.18 mg/kg), and folic acid (1.53 mg/kg). The enrichment of the flour was carried out according to a technical reference, developed by a National Technical Committee, respecting an enrichment process with international standards (22).

Morocco has mandatory fortification of industrial flour with Iron NaFeEDTA and folic acid. The wheat production in Morocco includes common wheat and durum wheat. The latter is typically used for a vast range of traditional foods such as pasta.

The small mills in rural and peri-urban areas tend to have a significant market share. The flour from the small mills is not fortified but it is used for traditional flat breads. The large mills import bread wheat mainly from USA, Canada, and Europe. The flour from the large mills is baked into French style and pan breads. The large mills have to follow mandatory flour fortification standards whereas the small mills do not fortify. The lack of fortification from the small milling sector (estimated at 35%−40% national market share) is a significant factor when it comes to closing the gap from 60%−65% of flour fortified to reaching the 90%−95% flour ideal level. Progress with getting the small mills for fortify is as challenging as the small maize mills in Sub-Saharan Africa.

Following the Morocco National Program to Combat Disorders Due to Micronutrient Deficiencies, a new premix has been proposed including electrolytic elemental iron (45 mg/kg of flour), vitamins B1 (4.5 mg/kg), B2 (2.79 mg/kg), B3 (36.18 mg/kg), and folic acid (1.53 mg/kg) (23).

The characteristics that soft wheat flours enriched with an iron-vitamin compound must meet in accordance with the provisions of article 5 of the above-mentioned decree n°2-19-144 of 8 Kaada 1440 (24) are as follows. First, the proportions of the constituents of one kilogram of the iron-vitamin compound premix “NaFeEDTA/folic acid,” used for the enrichment of the flours concerned include (a) NaFeEDTA (320 grams), (b) folic acid (4 grams), (c) fillers (starch, 676 grams). Second, the iron-vitamin compound “NaFeEDTA-folic acid” indicated above must be incorporated at the rate of 250 grams per ton of soft wheat flour concerned. The soft wheat flour enriched with the iron-vitamin compound “NaFeEDTA-folic acid” obtained must contain between 16.9 mg and 32.7 mg of iron per kg.

In a national survey on household consumption and expenditure (25–27), the consumption rates showed to be 185 kg/per year for cereal consumption in 2014, whereas the in 2001 the consumption rate was 185.2 kg/per year. This corresponds to 506 g of cereals consumed per person per day in 2014 (in g). Based on a daily consumption of 365.75 g of flour fortified with 10 mg/Kg of Iron NaFeEDTA, the Iron intake is 3.65 mg/day, or 38.4% of the EAR (estimated average requirements) women of childbearing age.

### Kingdom of Bahrain

The Ministry of Health of the Kingdom of Bahrain launched a national flour fortification programme to fortify flour with iron and folic acid in 2001. It launched as a national strategy and the aim of launching was to prevent and control iron deficiency, anemia, and neural tube defects among all age groups, especially children and women of reproductive age 15–49 years. In 2002, legislation to fortify flour with 1.5-ppm folic acid and 60-ppm iron was issued by the Ministry of Commerce and Industry. The kingdom of Bahrain Flour Mills Company has complied with the legislation. Since 2001, the Ministry of Health has been regularly monitoring and evaluating the fortification programme by collecting samples for laboratory analyzes. The program has had a positive impact on anemia prevalence among all age groups. During the past 18 years, the prevalence of anemia among women of reproductive age has decreased from 51.3% to 35.4%. In children of 9 months old, it has decreased from 58.2% to 41%. In addition, the neural tube defects have decreased from 25/10.000 live births in 2000 to < 5/10.000 live births in 2010.

## Current status on neural tube defects prevalence estimates in EMR

The global NTD prevalence is estimated to range from 15.3 to 23 (point estimate 18.6) per 10,000 live births (2). This implies an estimated 214,000 to 322,000 affected pregnancies annually.

The estimated prevalence of NTDs for countries in the EMR is 1.0 to 3.3/1,000 live births suffer from neural tube defects (17, 18). The lowest estimates were observed in Tunisia (1.6 per 10,000) and the highest in Iraq (269.6 per 10,000) (28) (Table 4). Currently, no systematic literature review has been published to provide a trend analysis or a good overview of current NTD prevalence estimates in the region.

**Table 4:** Overview NTD prevalence estimates in EMR.

**Country**	**National / Regional**	**Authors**	**Study year**	**NTD rates Per 10,000 Livebirths**	**Number of NTDs**	**Total number of (live) births**
Afghanistan	Regional	Hamed et al.	2016	Estimated 23–26	27	10,493
Bahrain	Regional	Al Arrayet et al.	2000	9.5	–	–
Djibouti	–	–	–	–	–	–
Egypt	Regional	Mohammed et al.	2007	16	8	5,000
Egypt	Regional	Shawky et al.	1966–2009	13.78	910	660,280
Iran	Regional	Abdirad et al.	2001–2005	25.7	36	13,997
Iran	Regional	Behrooz and Gorjizadeh	2002–2004	42	56	13,262
Iran	Regional	Delshad et al.	2005–2007	10.1	62	61,112
Iran	Regional	Ebrahimi, Esfahani, and Bagheri	2005–2011	48	68	14,034
Iran	Regional	TRoCA registry in ICBDSR Report 2014	2012	3.25	2	6,093
Iraq	Regional	Mahmood et al.	2002–2004	269.6	62	2,300
Iraq	Regional	Al-Obaidi et al.	2009	40.1	6	1,494
Jordan	Regional	Masri	1993–2002	11	119	28,301
Jordan	Regional	Aqrabawi	2002–2003	64.86	33	5,088
Jordan	Regional	Amarin and Obeidat	2005–2006	9.5	10	10,241
Jordan	Regional	Al-Qudah et al.	2005–2007	14	17	11,852
Kuwait	Regional	Madi et al.	2000–2001	6.5	5	7,739
Lebanon	National	Al Noaimi et al.	2014–2017	115.82	73	2,101
Libya	Regional	Singh and Al Sudani	1995	8	13	15,938
Morocco	Regional	Radouani et al.	2008–2011	13.3	80	60,017
Morocco	Regional	Forci et al.	2011–2016	10	44	43,923
Oman	National	Alasfoor and ElSayed	2013	23.2	139	60,000
Pakistan	Regional	Bokhari and Qureshi	2019–2020	260	52	2,000
Pakistan	Regional	Perveen and Tyyab	2000–2005	50.2	29	5,776
Pakistan	Regional (editorial)	Jooma	2002	16.7	16	9,580
Pakistan	Regional	Khattak et al.	2007	124.1	69	5,560
Pakistan	Regional	Qazi et al.	2009	68.8	35	5,082
Qatar	Regional	Bener et al.	1985–2009	10.9	311	302,049
Saudi Arabia	Regional	Al-Jama	1992–1997	53.5	79	14,762
Saudi Arabia	Regional	Asindi and Al-Shehri	1995–1998	7.5	64	82,176
Saudi Arabia	Regional	Murshid	1996–1997	10.9	18	16,514
Saudi Arabia	Regional	Seidahmed et al.	1996–2009	12	103	85,672
Saudi Arabia	Regional	Safdar et al.	1997–2005	12.5	42	33,489
Saudi Arabia	Regional	Al Rakaf et al.	2010–2013	17.3	49	28,376
Sudan	Regional	Omer et al.	2014–2015	28	103	36,785
Sudan	Regional	Satti et al.	2017	36	27	20,000
Syria	–	–	–	–	–	–
Tunisia	National	Nasri et al.	2008–2011	1.6	123	764,431
United Arab Emirates	Regional	Al Hosani et al.	1999–2001	2.1	–	–
Yemen	Regional	Dawood	2009	55.1	66	11,986

In this table, the NTD prevalence estimates are obtained from Chauhan et al. (28).

From a recent mapping study into the surveillance of birth defects in the EMR countries commissioned by the WHO-EMRO showed that Bahrain, Iran, Lebanon, Oman, and United Arab Emirates (UAE) have a national accurate population-based birth defect surveillance programme (BDSP) and the remaining 17 countries lacked a BDSP (29).

This shows the severe limitation of good surveillance data on NTDs. It is worthwhile to look back at each country to understand (i) how NTDs are monitored; (ii) comparison of surveillance programmes between countries (who has good systems to map NTDs, who doesn't). Based on this country examples can be provided who have good registries vs. countries who are in the process of setting up good registries.

To better illustrate the current status on the NTD prevalence in the EMR, a trend analysis from two countries Oman and Morocco will now follow.

### Example trend analysis of NTDs in Oman

**Figure 1:**
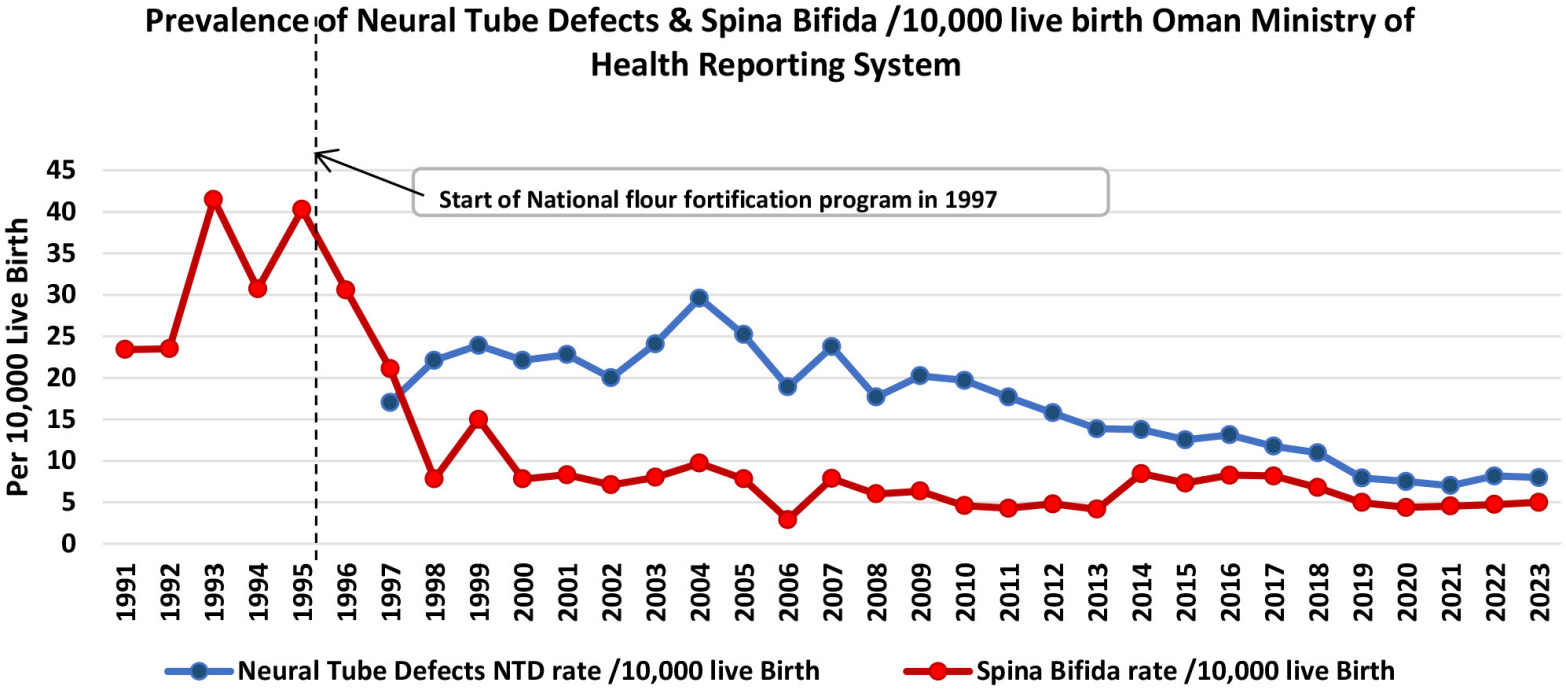
Trend analysis of neural tube defects and spina bifida prevalence estimated per 10,000 live birth in Oman 1991–2023.

The annual prevalence of congenital anomalies and NTD for the years 1991–2006 was published in 2010, based on the Omani annual health statistics reports shown in Table 5 and Figure 1 (13). It shows the changes in these rates since 1990, when the folate supplementation for pregnant women was started, and since 1997, when flour fortification was begun. There was no clear pattern observed in the prevalence of spina bifida between 1991 and 1996, fluctuating from 2.34 to 4.03 per 1,000 deliveries (23.5–40.3 per 10,000 deliveries), whereas after this period a sharp decline was observed, with the rate of spina bifida falling from 3.06 per 1,000 deliveries in 1996 to 2.11 in 1997 (30.6 to 21.1 per 10,000), a 31% reduction (13). The downward trend continued and by 2006 the prevalence was 0.29 per 1,000 deliveries (29 per 10,000 deliveries), an 88% reduction on the 1996 prevalence. The annual prevalence of other NTD per 1,000 deliveries did not show the same downward trend over the period that they were recorded (1997–2006) (13). On the other hand, the trend continues to decrease for NTD from 2006 till 2023 reaching 0.8 per 1,000 live birth (8 per 10,000 deliveries).

**Table 5:** Overview of NTD prevalence estimates in Oman 2014–2023.

**Year**	**NTD cases^a^**	**Total live birth**	**NTD prevalence^b^**	**NTD standard error (SE)**	**NTD 95% CI**	**Spina bifida cases**	**Spina bifida prevalence /10k**	**Spina bifida standard error (SE)**	**Spina bifida 95% CI**
2014	114	82,815	13.8	1.29	11.3–16.2	70	8.5	1.01	6.5–10.5
2015	108	86,296	12.5	1.2	10.2–14.9	63	7.3	0.92	5.5– 9.1
2016	116	88,448	13.1	1.22	10.7– 15.5	73	8.3	0.97	6.4–10.2
2017	105	89,330	11.8	1.15	9.5– 14.1	73	8.2	0.97	6.3–10.1
2018	94	85,696	11	1.12	8.9– 13.2	58	6.8	0.88	5.1–8.6
2019	67	84,530	7.9	0.86	6.2– 9.6	42	5	0.77	3.5–6.5
2020	62	82,578	7.5	0.83	5.9– 9.1	36	4.4	0.73	3.0–5.6
2021	57	81,434	7	0.78	5.5– 8.5	37	4.5	0.74	3.1–5.9
2022	62	76,037	8.2	0.85	6.5– 9.9	36	4.7	0.73	3.3–6.1
2023	56	70,285	8	0.95	6.1–9.9	35	5	0.74	3.5–6.5

^a^Number of cases, ^b^Prevalence rate per 10,000 live birth.

NTD, neural tube defects.

Oman is one country which shows an example of the success of the mandatory fortification folic acid which started since 1997 in reducing the cases of spina bifida and neural tube defects to lesser extent. The decline in spina bifida rate which reach 84% in 2023 from previous rate before fortification confirms the effects is due to fortification rather than supplementation which started since 1990 without any pattern changes in spina bifida rate. Through this national supplementation programme all pregnant women received iron and folic acid tablets at the first visit to the health center. An assessment of the programme in 1993 revealed that 97% of all pregnant women attending health centers received the supplements and the compliance rate adherence among them was estimated to be 77% (13). The reason of no effect of supplementation programme could be because the intake of the folic acid is only after registration of pregnancy in primary health care which usually happen late at the second or third month of pregnancy and not started before pregnancy as advised by WHO. The reduction in neural tube defects is less but still significant by 53% in 2023 from previous rate before fortification (Figure 1). The success of implementation of fortification since 1997 was supported by the political well lead by the Minister of Health who took the resolution to start it as mandatory legislation in the time USA was implemented it as subnational level. On the other hand, the fast application of the law was done because of the feasibility of the process which do not require additional equipment, supplies or manpower besides procurement of the fortificant. Oman Flour Mill which has 80% market share at that time is well equipped with modern technology which allow for swift application and quality monitoring of the process. This is in addition to the low cost of fortification which make this intervention cost effective one. There were no obstacles reported in this process which makes fortification continue to date and tolerant to additional fortificant as added in 2022 decree. This have to be supported by a monitoring process which shared between relevant stakeholder because of the map of the market share changes over the years and many new mills appear in the market. There are also the small-scale mills which despite its contribution is limited to the market but in doubt if they practice the fortification because of the technical limitation.

The experience of Oman shows also the importance of mandatory fortification rather than voluntary fortification which was supported by the research evidence. For example, a systematic review and metanalysis conducted in 2016 included 179 studies in the systematic review and 123 in a meta-analysis for the studies between January 1985 and December 2010. Result showed that in studies of live births (LBs) alone, period prevalence of spina bifida was lower in geographical regions with mandatory (33.86 per 100,000 LBs) vs. voluntary (48.35 per 100,000 LBs) folic acid fortification, and lower in studies of LBs, stillbirths, and terminations of pregnancy in regions with mandatory (35.22 per 100,000 LBs) vs. voluntary (52.29 per 100,000 LBs) fortification. In LBs, stillbirths, and terminations of pregnancy studies, the lowest pooled prevalence estimate was in North America (38.70 per 100,000). This can be explained because Canada and the United States were the first countries to require mandatory fortification and multiple studies have documented a pre–post reduction in neural tube defects (30, 31).

### Example trend analysis of NTDs in Morocco

An inspection of data from 20 public hospitals in Morocco (as described in the method section) was made for two separate periods (2012–2014 and 2017–2022). The main reason is a notable change in the periods 2012–2014 and 2017–2022 are: (i) The national distribution of number of regions decreased from 16 regions to 12 regions; and (ii) migration from a manual NTDs reporting system to one included in the national maternal and child health information system. See also Table 6 (the period 2012–2014 is available in the Supplementary Table 1).

**Table 6:** Regional distribution of neural tube defects rates in Morocco 2017–2022.

**Region**	**Spina bifida^a^**	**Rate of Spina bifida^b^**	**Anencephalia^a^**	**Rate of anencephalia^b^**	**NTD^a^**	**Rate of NTD^b^**
Tanger-Tetouan-Al Hoceima	123	4.32	145	5.09	268	9.41
Oriental	185	12.22	57	3.76	242	15.98
Fès-Meknès	147	4.20	145	4.14	292	8.34
Rabat-Salé-Kénitra	86	2.35	79	2.15	165	4.50
Béni Mellal-Khénifra	146	7.77	107	5.70	253	13.47
Grand Casablanca-Settat	115	2.73	79	1.88	194	4.61
Marrakech-Safi	140	3.70	102	2.69	242	6.39
Drâa-Tafilalet	72	5.02	96	6.70	168	11.72
Souss-Massa	97	5.08	123	6.44	220	11.52
Guelmim-Oued Noun	12	3.08	18	4,62	30	7.70
Laayoune-Sakia El Hamra	11	2.75	21	5.24	32	7.99
Eddakhla-Oued Eddahab	5	3.20	5	3.20	10	6.40
Total	1,139	4.43	977	3.80	2,116	8.23

^a^Number of cases, ^b^Rate per 10,000 live birth.

NTD, neural tube defects.

Data for the period 2012 to 2014 comes from the maternity hospitals of the 20 Moroccan hospitals in the study. Notification registers have been set up for this purpose, with a predefined circuit, training of the healthcare providers who provide the notification and a monthly transfer of the data thus compiled to The Nutrition Cell of the Population Department of the Ministry of Health which makes the treatment.

**Figure 2:**
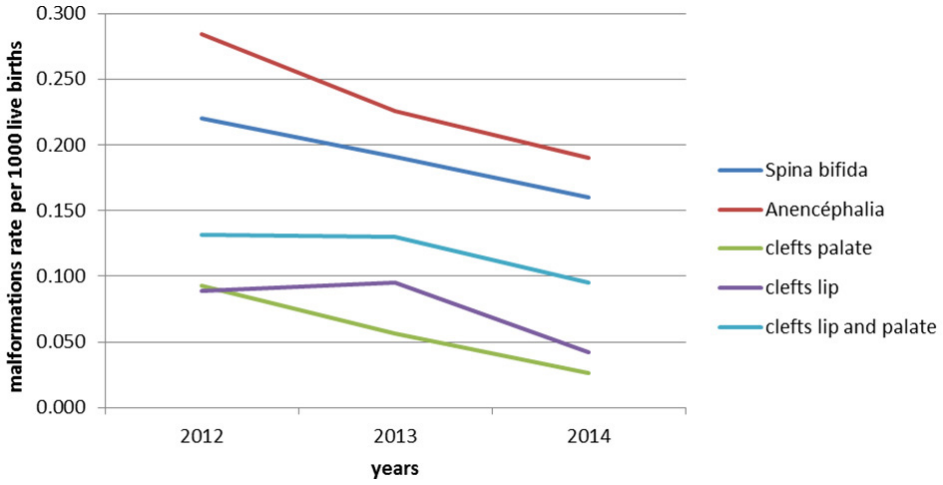
Prevalence estimates per 1,000 live births for each congenital malformation recorded annually in the maternity hospitals of the 20 public hospitals in Morocco during the period 2012–2014.

The analysis of rates' evolution (per 1,000 births) of malformations recorded annually of neural tube and oro-facial clefts (see also Figure 2) revealed a significant decrease in rates of spina bifida, anencephaly, palate clefts, labial clefts, and Labio-palatam clefts (*p* = 0.01). The analysis of the overall data also revealed a significant decrease in the prevalence of neural tube closure anomalies and oro-facial clefts during the period of 2012–2014. Indeed, annual anomalies of neural tube closures rates and oro-facial clefts decreased, respectively from 5.05 to 3.5/10,000 and 3.13 to 1.64/10,000 live births, which was statistically significant (*p* = 0.01).

Data from the years 2017 to 2022 were obtained from the new system in monitoring birth defects in Morocco. Among the new components that have been integrated into the new system, the registrations of neural tube defects can be found. The generalization of the new revised information system to the regions was the subject of Circular No. 122/DPRF/DPE published on October 12, 2015, in order to standardize the entry and processing of data at all levels. Despite the multiple advantages cited above, this transition between the old and the new MCH/PF application has impacted the availability of data, especially as it is the subject of a parallel notification system as is the case for the notification of NTD, particularly in the year of the 2015–2016 revision.

**Figure 3:**
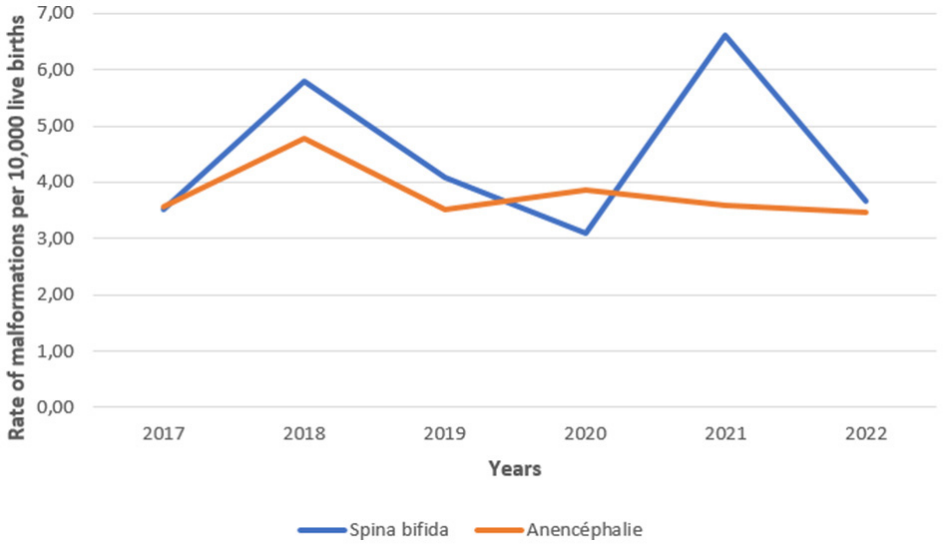
Evolution of the total cases of neural tube defects recorded annually in Morocco during the period 2017–2022.

**Figure 4:**
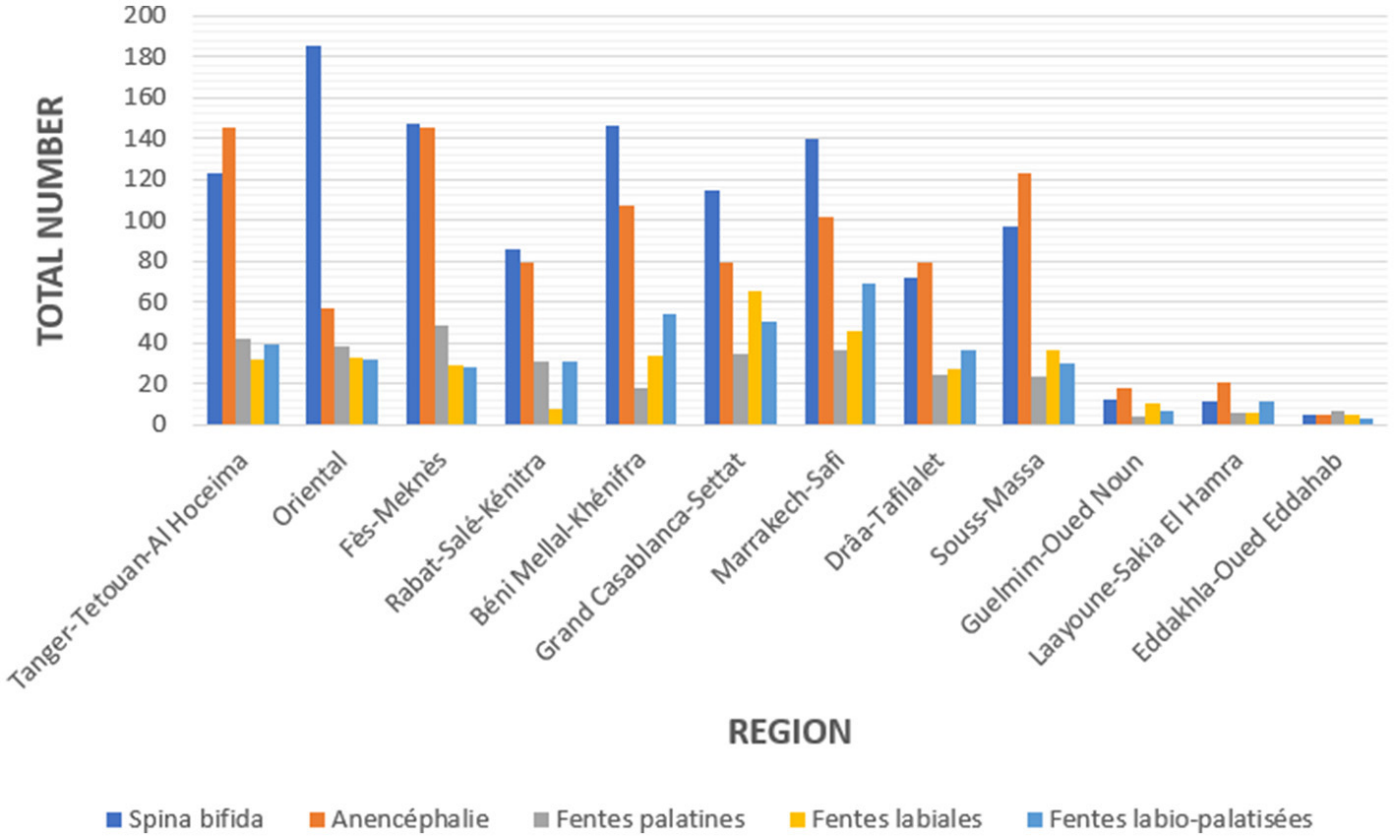
Regional distribution of the number of cases of malformations recorded in Morocco during the period 2017–2022.

A total of 2,569,763 live births were recorded at reporting sites during the period 2017–2022. From all livebirths in this period, a total of 3,180 malformations were detected. From these, 116 (66.54%) corresponds to cases of neural tube defects (NTD) and 1,064 (33.46%) to cases of orofacial clefts (OFC), with respective rates of 8.23 and 4.14 per 10,000 live births. The overall rate of all malformations is 12.37 per 10,000 live births. The Figures 3, 4 below visualize the distribution of these anomalies. NTDs represent the most predominant anomalies in our study. The most frequent malformations in our study were Spina bifida for NTDs and cleft lip-palate for orofacial clefts.

From the data, it was shown through the analysis of the results of the first period in which data collection was manual that the prevalence of NTDs compared to the period before 2011 has decreased significantly but it is lower than the period 2017 to 2022. This observation could be explained by the change in the notification system which has become more responsive and precise. The migration from a manual reporting system to a computerized system resulted in the loss of information for 2 years, 2015 and 2016.

Importantly, the analysis show a decrease of prevalence from 21.73 cases per 10,000 live births in 2008 to 8.23 per 10,000 live births over the last years analyzed. Over the same period, the prevalence of folic acid deficiency among women of childbearing age has also decreased significantly. Interestingly, the prevalence of lip-palatal anomalies follows the same trends.

From the data of the last period regional disparities can be observed. The four regions maintain high prevalence compared to the rest of the national territory. But the information system does not allow these prevalences to be correlated with the consumption of fortified flour. The system also does not provide information on the gender of patients and the possible presence of consanguinity.

Another notable finding is the decline of declared cases in the period from 2017 to 2022, as well the prevalence of NTDs in 2019 and 2020. An explanation could be due to under-reporting but also probably by an under-use of maternal health services observed during this Covid-19 pandemic period. The further decline of the prevalence observed in 2022 suggests that these are probably not the only explanations.

## Conclusions

Food fortification of staple foods has proven to be an effective strategy in reducing the prevalence of NTDs, particularly in the EMR. This aligns with the WHO's long-standing recommendation that large-scale food fortification is a powerful and cost-effective public health measure to address micronutrient deficiencies in populations.

Countries like Oman and Morocco have demonstrated significant improvements in NTD prevalence through mandatory food fortification programs, which often follow WHO guidelines on fortificants and levels. However, several common challenges and lessons learned have emerged, echoing WHO's emphasis on the need for careful monitoring, enforcement, and public education to ensure these programs achieve their intended impact.

The EMR faces considerable challenges in addressing micronutrient deficiencies and improving public health. A primary issue is the lack of data on micronutrient status and the prevalence of NTDs. This data gap hinders effective policy-making and monitoring of progress. In addition, the monitoring systems for wheat flour fortification programs at mills are inadequate, which has impeded the effectiveness of fortification initiatives in the region. Another significant concern is that seven countries in the region—Tunisia, Libya, Lebanon, Egypt, Pakistan, Syria, and Somalia—have yet to implement flour fortification programs, worsening the problem. Furthermore, the lack of regional data on the cost-effectiveness of flour fortification programs is a critical issue, even though available evidence shows that fortification can reduce iron deficiency, iron deficiency anemia, and NTDs, thereby lowering the economic burden on families. There is also a notable insufficiency in the implementation of supplementation programs, which further limits efforts to combat micronutrient deficiencies (32).

To enhance the effectiveness of fortification programs and improve the monitoring of birth defects, several actionable points have been recommended by the WHO (33):

1. Establish National Registries: Establish registries at maternity hospitals and public health centers to track birth defects and related health data.
2. Conduct National Surveys: Carry out national surveys regularly to monitor progress, identify gaps, and assess the impact of fortification efforts.
3. Strengthen Data Collection and Monitoring: Improve systems for data gathering and continuous monitoring to ensure accurate and reliable information.
4. Enforce Monitoring Systems: Implement stricter oversight and monitoring of food fortification programs at mills to ensure proper execution and targeting of fortified foods.
5. Build Capacity and Political Commitment: Train mill workers, managers, and inspectors to enhance the technical capabilities and ensure proper fortification.
6. Foster Multisectoral Collaboration: Encourage collaboration across various sectors and ministries to create a unified approach to health and nutrition.
7. Provide Financial and Technical Support: Offer financial and technical support to countries to strengthen their fortification and health initiatives.
8. Implement Research and Advocacy Initiatives: Support research to provide evidence-based solutions and raise awareness through advocacy campaigns.
9. Address Social Determinants of Anemia: Implement community-level interventions that address both nutritional and non-nutritional causes of anemia through integrated planning and social support.

A regional assessment of wheat flour fortification in the EMR (32) has emphasized key recommendations for optimizing fortification efforts. These include:

1. Nutritional Needs and Deficiencies: National decision-makers should consider the prevalent nutrient deficiencies in their populations when deciding what vitamins and minerals to add to flour.
2. Flour Consumption Profile: Understanding the total amount of flour produced and consumed in each country, including both domestically milled and imported flour, is essential to determine the feasibility and scope of fortification.
3. Sensory and Physical Effects: The impact of fortification on the texture, taste, and appearance of flour products should be taken into account to ensure consumer acceptance.
4. Fortification of Other Foods: Consideration should be given to fortification efforts in other foods to avoid over-fortification and ensure nutrients are appropriately balanced.
5. Use of Supplements: The current consumption of vitamin and mineral supplements by the population should be factored in when deciding on fortification.
6. Costs: The financial feasibility and cost-effectiveness of fortification measures must be carefully evaluated to ensure that fortification programs are sustainable and impactful.

In conclusion, NTDs are a preventable global health issue that continues to affect communities worldwide. The experiences of countries like Oman and Morocco demonstrate the effectiveness of folic acid food fortification in significantly reducing the prevalence of NTDs. While the EMR has made substantial progress in promoting food fortification, more work is needed to expand its coverage and enhance its effectiveness. Addressing the challenges outlined, such as improving data collection, monitoring, and multisectoral collaboration, will be critical for achieving the desired public health outcomes in line with WHO recommendations.

## Data Availability

The original contributions presented in the study are included in the article/Supplementary material, further inquiries can be directed to the corresponding author.
