# Supplementary material for: Mandatory food fortification in the eastern Mediterranean region results in reduced prevalence of neural tube defects
Source: Front Public Health. 2026 Jan 12;13:1664607. doi: 10.3389/fpubh.2025.1664607 (PMC12832505; doi:10.3389/fpubh.2025.1664607)
Supplement: Supplementary file 1 [file Table_1.docx]

**Suppl. Table 1:** Regional distribution of Neural Tube Defects Rates in the maternity wards of 20 public hospitals, Morocco, 2012-2014

| **Region** | **Spina bifida^a^** | **Rate of Spina bifida ^b^** | **anencephalia^a^** | **Rate of anencephalia** | **NTD^a^** | **Rate of NTD^b^** |
| --- | --- | --- | --- | --- | --- | --- |
| Oued Eddahab-Lagouira | 1 | 11.21 | 0 | 0 | 1 | 11.21 |
| Laâyoune-Boujdour-Sakia Lhamra | 2 | 1.45 | 10 | 7.25 | 12 | 8.70 |
| Guelmim-Smara | 0 | 0.00 | 2 | 1.54 | 2 | 1.67 |
| Souss-Massa-Darâa | 5 | 0.60 | 11 | 1.32 | 16 | 1.92 |
| El Gharb-Chrarda-Bni Hssen | 14 | 2.31 | 30 | 4.95 | 44 | 7.27 |
| Chaouia-Ouardigha | 2 | 0.61 | 2 | 0.61 | 4 | 1.21 |
| Marrakech-Tensift-El Haouz | 22 | 2.14 | 19 | 1.85 | 41 | 4.07 |
| Oriental | 10 | 1.56 | 9 | 1.40 | 19 | 2.96 |
| Grand Casablanca | 9 | 1.39 | 8 | 1.23 | 17 | 2.70 |
| Rabat-Salé-Zemmour-Zaër | 4 | 1.09 | 11 | 3.00 | 15 | 4.08 |
| Doukkala – Abda | 35 | 5.65 | 14 | 2.26 | 49 | 7.90 |
| Tadla-Azilal | 16 | 2.93 | 15 | 2.74 | 31 | 5.99 |
| Meknes-Tafilalet | 14 | 1.94 | 16 | 2.22 | 30 | 4.25 |
| Fes-Boulemane | 3 | 1.94 | 3 | 1.94 | 6 | 4.29 |
| Taza-Al Hoceima-Taounate | 4 | 1.50 | 17 | 6.37 | 21 | 7.87 |
| Tanger-Tetouan | 7 | 0.99 | 15 | 2.13 | 22 | 3.12 |
| total | 148 | 1.91 | 182 | 2,35 | 330 | 4.32 |

^a^ number of cases , ^b^ rate per 10 000 live birth, NTD : neural tube defects
